# Ion Channel and Transporter Involvement in Chemotherapy-Induced Peripheral Neurotoxicity

**DOI:** 10.3390/ijms25126552

**Published:** 2024-06-14

**Authors:** Eleonora Pozzi, Giulia Terribile, Laura Cherchi, Sara Di Girolamo, Giulio Sancini, Paola Alberti

**Affiliations:** 1 Experimental Neurology Unit, School of Medicine and Surgery, University of Milano-Bicocca, 20900 Monza, Italy; eleonora.pozzi@unimib.it (E.P.); l.cherchi1@campus.unimib.it (L.C.); sara.digirolamo@unimib.it (S.D.G.); 2 Human Physiology Unit, School of Medicine and Surgery, University of Milano-Bicocca, 20900 Monza, Italy; giulia.terribile@unimib.it (G.T.); giulio.sancini@unimib.it (G.S.); 3 Fondazione IRCCS San Gerardo dei Tintori, 20900 Monza, Italy

**Keywords:** chemotherapy-induced peripheral neurotoxicity, chemotherapy-induced peripheral neuropathy, neuropathic pain, ion channels, neuropathy, NCX, sodium voltage-operated channels, axonal damage, potassium channels

## Abstract

The peripheral nervous system can encounter alterations due to exposure to some of the most commonly used anticancer drugs (platinum drugs, taxanes, vinca alkaloids, proteasome inhibitors, thalidomide), the so-called chemotherapy-induced peripheral neurotoxicity (CIPN). CIPN can be long-lasting or even permanent, and it is detrimental for the quality of life of cancer survivors, being associated with persistent disturbances such as sensory loss and neuropathic pain at limb extremities due to a mostly sensory axonal polyneuropathy/neuronopathy. In the state of the art, there is no efficacious preventive/curative treatment for this condition. Among the reasons for this unmet clinical and scientific need, there is an uncomplete knowledge of the pathogenetic mechanisms. Ion channels and transporters are pivotal elements in both the central and peripheral nervous system, and there is a growing body of literature suggesting that they might play a role in CIPN development. In this review, we first describe the biophysical properties of these targets and then report existing data for the involvement of ion channels and transporters in CIPN, thus paving the way for new approaches/druggable targets to cure and/or prevent CIPN.

## 1. Introduction

Chemotherapy-induced peripheral neurotoxicity (CIPN) is a relevant, potentially persistent adverse event of the most commonly used drugs in cancer treatment: platinum drugs, taxanes, vinca alkaloids, proteasome inhibitors and thalidomide [1]. CIPN features are mainly ones of sensory polyneuropathy, even if motor and autonomic impairment is also reported [1]. Sensory disturbances at limb extremities consist of paresthesia/dysesthesia and neuropathic pain at limb extremities and a sensory loss that can be so pronounced that it impairs fine manipulation and gait [2].

In the state of the art, there is no curative or symptomatic treatment for this condition, even though a moderate effect of duloxetine as a symptomatic treatment was demonstrated [3]. An incomplete knowledge of CIPN mechanisms is one of the main reasons for this unmet clinical need. In the last few years, (an) intriguing target(s) emerged as potentially relevant for preventing/treating CIPN: modulation of ion channels/transporters. In this review, we provide first a description of the potential target of interest, and we report studies describing their potential involvement in CIPN.

## 2. Biophysical Properties of Transporters/Ion Channels of Interest

**Figure 1:**
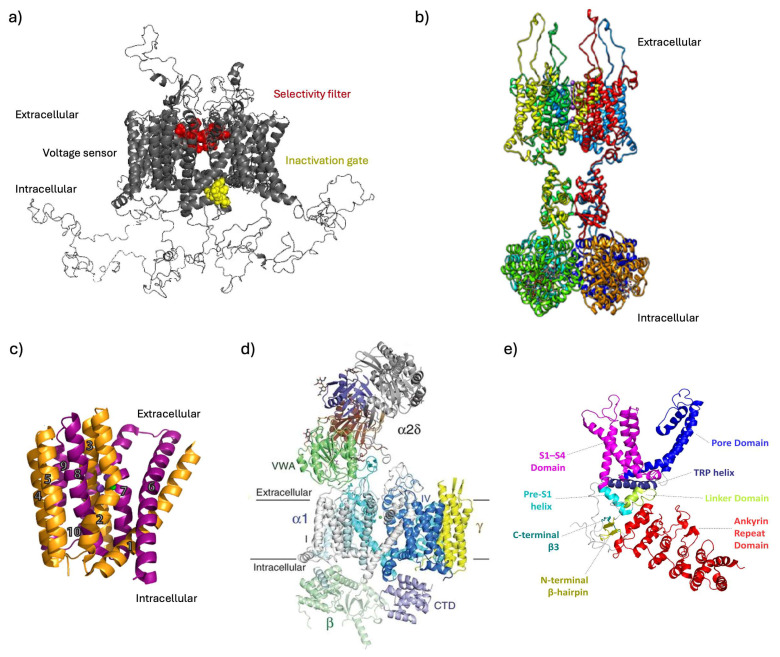
Channels’ 3D structures. (**a**) 3D structure of voltage-gated Na^+^ 1-type channel (Homo sapiens). The scheme includes all domains: the selectivity filter (red), the inactivation gate (yellow) and the voltage sensor (gray). Modified from Romanova et al., 2022 [4]. (**b**) 3D structure of voltage-gated K^+^ 1-type channel. View from the membrane (side view). Two of four domains are shown, with front and back domains deleted to allow the structure to be seen. Purple spheres represent K^+^ ions. Modified from Kariev et al., 2024 [5]. (**c**) 3D structure of sodium–calcium exchanger (Methanococcus jannaschii). Helices 1–5 (TM1–5) are orange, and helices 6–10 (TM6–10) are purple. Purple and green spheres represent Na^+^ and Ca^2+^ ions, respectively. Modified from Giladi et al., 2016 [6]. (**d**) Three-dimensional structure of high-voltage-activated Ca^2+^ channel (rabbit). The structure model is color-coded for distinct subunits. Green sphere represents Ca^2+^ ion. Modified from Mochida et al., 2018 [7]. (**e**) 3D structure of TRPV3 (mouse). Side view of the tetramer protein. The structure model is color-coded for distinct subunits. Modified from Kalinovskii et al., 2023 [8].

In Figure 1, the general features of each class of transporters/ion channels addressed are presented.

### 2.1. Voltage-Gated Na^+^ Channels (VGSCs)

Voltage-gated Sodium (Na^+^) channels (VGSCs) play a strategical role in pain mechanisms as they are involved in the cellular excitability and, not only in the generation, but also in the propagation, of action potential (AP). AP is the main unit for conducting information, including painful stimuli from the peripheral receptors to the higher centers [9]. VGSCs are considered key determinants of nociceptor excitability [10]. Clinical and experimental data suggest that changes in VGSC expression [11], trafficking or kinetic properties [12] could play a key role in the pathogenesis of neuropathic pain. Indeed, they have been studied to investigate the aetiology of pathological pain sensation, and they have been considered important pharmacological targets [13].

#### 2.1.1. Topology and Gating

VGSCs are heteromeric transmembrane complexes consisting of a principal highly glycosylated [14] pore-forming α subunit (260 kDa), sufficient on its own to form a functional ion-conducting voltage-gated channel, and an additional β subunit (33–45 kDa) involved (one or more) in the modulation of channel gating. The α subunit is constituted by four homologous (with amino acid homology > 75% [14]) domains (D1–D4) connected to each other by three intracellular loops, respectively (L1–L3). Each domain consists of six transmembrane-spanning hydrophobic α-helical segments (S1–S6), and each segment is connected by two intracellular (between S2–S3 and S4–S5) and three extracellular linkers (between S1–S2, S3–S4 and S5–S6; the last ones are called P-loops). Both the N-terminus and C-terminus are intracellular [9].

Among segments, S4 is highly conserved and acts in each domain as a voltage sensor for the channel. This region contains a net electrical charge, called a gating charge, due to the presence of basic amino acids (positively charged, i.e., lysine and arginine). The movement of these positive charges through the electrical field, in response to a change in the membrane potential, imparts a change in free energy, needed for the transition of the channel between its functional states (called gating) [15]. Indeed, the channel can assume three different functional states: (i) closed and activatable (resting), (ii) open (active) and (iii) closed and non-activatable (inactivated or refractory) [13]. VGSCs stay in the closed state under membrane resting potentials, they open on depolarization and they close rapidly (fast inactivation) on repolarization, or more slowly (slow inactivation) on prolonged depolarization. This process leaves the channel refractory for some time after repolarization, and it has to recover back to the closed state to open again on the next depolarization. Recovery after inactivation is called repriming [16]. This inactivation is known as open-state inactivation. There is another type of inactivation, known as closed-state inactivation, that occurs when a channel can inactivate directly from a closed state before it opens. The two types of inactivation are not mutually exclusive, although many channels inactivate mainly through one of these [17]. Some VGSCs (i.e., most of the VGSCs of Purkinje cells) open with depolarization, but then, rather than inactivating, they become blocked due to an open channel blocker. In this case, blocked channels reopen upon repolarization as the blocker unbinds, producing the so-called resurgent current [18].

Although resurgent current flows through the same channels as transient (in the voltage clamp: brief macroscopic Na^+^ current evoked by a depolarization step) and persistent current (the proportionately tiny, residual Na^+^ current that lasts throughout the step), it shows different voltage dependence and kinetics. It is dynamically gating (differently from persistent current), and, at any given potential, it has a rising phase, a peak and a falling phase [18].

According to the molecular mechanism underlying the gating properties of VGSCs, on depolarization, positive charges within S4 move outward along a spiral path, initiating a morphological change that opens the pore, allowing the inward passage of Na^+^ (rising phase of the AP). The movement toward the extracellular side is accompanied by the movement of the linker L3 (DIII–DIV), which acts as an “inactivation gate”. This movement is probably due to a reduction in electrostatic repulsion between the charges in S4 and those in the linker, and it allows the process known as fast inactivation (on a timescale of ms) of the channel (involved in the termination of AP and in the regulation of the refractory period). On sustained depolarization, VGSCs undergo a process known as slow inactivation (on a timescale of s), involved in the regulation of membrane excitability by increasing the AP threshold and limiting their burst duration and their propagation within dendrites. After the refractory period, the recovery occurs when S4 goes back into the membrane, and the inactivation gate moves away from the pore [19].

According to the molecular mechanism underlying the Na^+^ influx upon repolarization, it has been suggested that endogenous factors, functioning as an open channel blocking particles (i.e., small intracellular peptides), can enter the channel while it is open. Upon repolarization, the blocker is expelled due to its positive charge, giving rise to resurgent currents [20].

Regarding the channel structure, among the linkers of the four domains, the most important are L3 (DIII–DIV) and L1 (DI–II). The first one contains three hydrophobic residues (isoleucine, phenylalanine and methionine) that are involved in the fast inactivation of the channel, and it is phosphorylated by protein kinase C (PKC); the second one is phosphorylated by protein kinase A (PKA) [21]. The phosphorylation may be a key element in the modulation of channel activity since PKC and PKA are involved in neuropathic and inflammatory pain, respectively [22].

Another important part of the receptor is the portion that connects the segments S5 and S6 (called P-loops), which contains four conserved amino acids, aspartate, glutamate, lysine and alanine (DEKA motif), that represent the selective filter which determines the channel’s Na^+^ permeability over the other cations [23].

#### 2.1.2. Classification and Isoforms Involved in Neuropathic Pain

There are nine functionally characterized α isoforms (Nav 1.1–1.9) and four β isoforms (β_1_–β_4_). The different isoforms are characterized by a different level of homology, as discussed in detail by Isom, an aspect that should be carefully weighted since [24] these isoforms display different kinetics and voltage-dependent properties, and they are associated with auxiliary protein that regulates channel trafficking and gating. These characteristics allow a cell-type-specific modulation of the channel [25].

In the absence of subtype-selective Na^+^ channel blockers, the isoforms are classified according to their sensitivity to tetrodotoxin (TTX) in TTX-sensitive ones (Na_v_ 1.1–Na_v_ 1.4, Nav 1.6 and Nav 1.7) that have an IC50 in the nanomolar range and TTX-resistant ones (Nav 1.5, Nav 1.8, Nav 1.9) that have an IC50 in the micromolar range [14].

Among the nine functionally expressed isoforms, four are considered to have a key role in the pathogenesis of neuropathic pain: Nav 1.3, Nav 1.7, Nav 1.8 and Nav 1.9 [13,25].

**Nav 1.3**

The Nav 1.3 isoform is encoded by the SCN3A gene, and it is a TTX-sensitive isoform (Kd = 1.8–4 nM) [25]. It is highly expressed in the central nervous system (CNS) [9]. This isoform produces a fast inactivation and activation current, and it is characterized by rapid repriming. It shows slow closed-state inactivation that leads to a large ramp current in response to small, slow depolarization. The regulation is cell dependent [26]. Nav 1.3 is upregulated in several pain disorders, and the resulting hyperexcitability may explain its involvement; the fast kinetic that characterizes this channel supports its role in allowing peripheral nerves to fire at high frequencies [27,28].

**Nav 1.7**

The Nav 1.7 isoform is encoded by the SCN9A gene, and it is a TTX-sensitive isoform (Kd = 4.3–25 nM) [25]. It is preferentially expressed in peripheral neurons and normally highly expressed in small-diameter dorsal root ganglion (DRG) neurons with unmyelinated and slow conduction axons (C-fibers) [13]. This channel is involved in the regulation of sensory neurons’ excitability, and it is one of the main contributors to human pain disorders [29].

It is characterized by a fast activation and inactivation but slow repriming; these biophysical properties make it well suited for low-frequency firing in C-fibers. Moreover, Nav 1.7 is characterized by slow closed-state inactivation, a mechanism that allows the channel to produce a large ramp current in response to small and slow depolarizations [30]. The ability of this isoform to boost subthreshold stimuli increases the probability that neurons can reach their threshold for firing APs. Based on these characteristics, Nav 1.7 is thought to act as a threshold channel [31]. This isoform produces resurgent currents in a subset of DRG neurons (their production crucially depends on cell background). These currents are triggered by repolarization following a strong depolarization and support burst firing in, for example, cerebellar Purkinje neurons [32]. Nav 1.7 channels might also regulate neurotransmitter release at the nociceptors’ central terminals [33].

The key role of the Nav 1.7 isoform in pain mechanisms is supported by the evidence that different mutations affecting the SCN9A gene can modify its biophysical properties in a pro-excitatory manner compared to the wild-type channel by (a) causing a hyperpolarization shift in activation, allowing the channel to open after weaker depolarization [34]; (b) causing wider ramp currents after the same small, slow depolarization [35]; (c) impairing slow activation with the result of increased firing rate [36]; (d) causing a depolarizing shift in fast inactivation, resulting in fewer inactivated channels at any given potential and a persistent current [37]; and (e) causing an increase in resurgent currents [37]. Moreover, some mutations cause a decrease in the single AP threshold and an increase in the firing frequency in small DRG [38].

**Nav 1.8**

The Na_v_ 1.8 isoform is encoded by the SCN10A gene, and it is a TTX-resistant isoform (Kd = 40–60 μM) [25]. It could represent an ideal therapeutic target because it is selectively expressed in sensory neurons and mostly in small-diameter DRG neurons [13]. This isoform is characterized by a slow activation and inactivation [13] but rapid repriming [25] and depolarized voltage dependency for activation and inactivation. It produces the majority of the Na^+^ current during the AP depolarizing phase in neurons in which it is expressed [39]. It can support repetitive firing in response to depolarizing input [10]. All these biophysical properties and its localization in free nerve endings suggest that the Nav 1.8 isoform may have an important role in nociceptor excitability [29] and in nociceptive information transmission [10].

**Nav 1.9**

The Nav 1.9 isoform is encoded by the SCN11A gene, and it is a TTX-resistant isoform (Kd = 40 μM) [25]. It is selectively and highly expressed in small-diameter DRG neurons with unmyelinated and slow conduction axons (C-fibers) [13], but it is downregulated in injured neurons [40]. This isoform is characterized by hyperpolarized voltage dependency of activation, close to the resting membrane potentials of neurons (−60/−70 mV), and it is characterized by ultraslow inactivation [30]. These properties allow it to produce a persistent Na^+^ current since its activation and inactivation curves allow it to be activated at resting potentials [41]. A downregulation in its expression and the consequent associated decrease in the persistent current could lead to more hyperpolarized membrane potentials, allowing recovery of TTX-sensitive Na^+^ channels from inactivation [40].

### 2.2. Voltage-Gated K^+^ Channels (Kv)

Since the first extraordinary landmark studies by Hodgkin and Huxley using the patch clamp technique, knowledge about voltage-dependent potassium channels is becoming ever deeper. K^+^ channels are probably the largest and most diverse family of ion channels [42,43,44], represented by more than 80 known loci, which encode multiple pore-forming subunits in the mammalian genome. To account for the expansion of identified K^+^ channel genes, a parallel—KCN—nomenclature was initiated by the Human Genome Organization (HUGO) [45], which complements the standardized—Kv—nomenclature.

#### 2.2.1. Topology and Gating

Among K^+^ channels, the most important contributors to neuronal excitability are the voltage-dependent Kv channels that regulate resting membrane potential, membrane repolarization, action potential shape, firing frequency and adaptation in both the central and the peripheral nervous system (CNS and PNS, respectively) [42,46].

The Kv channels are tetramers of α subunits, each with S1–S6 α-helical transmembrane segments. The S4 segment is the voltage sensor that allows the opening of the channel at a precise membrane potential (high- or low-threshold kinetics) depending on the specific channel type. This leads to the activation of an outward K^+^ ion current across the cell membrane via the S5–S6 pore (P-region) [47,48,49,50]. There are approximatively 40 mammalian genes encoding α subunits, which are divided into 12 families (Kv1–12) [43,47]. Different genes within a family are denoted with an additional number after the decimal point, such as Kv1.1 and Kv1.2, roughly in order of their molecular characterization [43]. Both the N- and C-termini are located intracellularly, and often link auxiliary citoplasmatic β subunits (Kvβ) that further modify the gating properties, the neuronal distribution and the functions of Kv channels [42,45,48]. An important example is represented by the Kvβ2 subunit, which strongly modifies the K1.1 and K1.5 channels’ activation threshold both directly and indirectly [43,48]. Moreover, the β subunit of Kvβ2.1 allows the distribution of these channels in the juxtaparanodal zones adjacent to nodes of Ranvier in large-diameter myelinated axons in both the CNS and the PNS [46,51].

It is now widely known that Kv channels form an exceedingly diverse group, much more so than one would predict simply based on the number of distinct genes that encode them. This diversity arises from several factors [44]. The main one is the ability to form both homo- and hetero-tetramers between different subunits within the same family. Each subunit’s composition could dictate distinct biophysical and functional properties, different interactions with second messengers, variable spatial and temporal expression and diverse regulation by pathophysiological processes [42,44,52]. Furthermore, the presence of a variety of modulatory partners can also critically modify Kv functions. Notably, members of the Kv5, Kv6, Kv8 and Kv9 families encode 10 “silent” subunits (KvS), which do not form conducting channels on their own but co-assemble with other Kv subunits with significant physiological consequences [44,52]. An important example is the association of Kv9.1 or Kv9.3 with Kv2.1, which leads to increased currents compared with Kv2.1 homomers. Moreover, the interplay of Kv2.1/Kv9.1 in A-fiber neurons allows the channel involvement in pain signaling and a direct participation in nociceptive pathways [52,53].

#### 2.2.2. Channel Kinetics

**Inactivating K^+^ Channels**

Among many, one reliable way of distinguishing the huge variety of K^+^ voltage-dependent channels is by current kinetics and, in particular, the presence or absence of inactivation. Regarding the inactivating group, many neurons express two main classes of currents: slow inactivating, commonly called D-current (I_D_), and fast inactivating or A-current (I_A_) [54,55,56]. I_D_ has been reported to differ from I_A_ in so far as it shows slower inactivation rates and enhanced sensitivity to K^+^ channel selective blockers [56,57]. Moreover, I_D_ has been identified in different types of neurons with a fast-conducting axon. Here, it provides a secure conduction, synchronizing rapid synaptic inputs and facilitating rapid membrane recovery [56,58].

Like I_D_, I_A_ is activated transiently at a low-threshold level but inactivated rapidly during both large and small depolarizations from rest. Thus, I_A_ has a fine mechanism that modulates its amplitude even with small voltage changes around the resting potential [55,59]. I_A_ is mediated primarily by Kv4 family α subunits, which are widely expressed in both the CNS and PNS. In particular, Kv4 mainly localizes in cell bodies and dendrites of cortical and hippocampal pyramidal neurons, in small nociceptive type neurons and in the larger mechanoreceptor type [56,60]. This specific neuronal distribution allows I_A_ not only to regulate the integration and the propagation of the excitatory synaptic potentials, but also to modulate the back-propagating potentials in dendritic branches [60,61]. Among the five A-channels in mammals, recently, several authors have focused their attention on two specific subunits: Kv4.2 and Kv4.3 [43,55,62,63]. The latter is often associated with different β subunits and multiple K^+^ channel interacting proteins, such as KChIPs, which can modulate Kv4 current properties, neuronal trafficking and, in turn, its functions [63,64]. Kv4.2 subunits are specifically localized in the dendritic membrane, fine-tuning the back-propagation of signals and dendritic excitability, whereas Kv4.3 resides principally on the soma [43,62]. Highlighting Kv4’s fundamental functions, different defects in trafficking, expression or kinetics are observed in several disorders, such as memory deficits or peripheral neuropathic pain [55,57,62].

**Delayed Rectifier Currents**

Regarding the non-inactivating K^+^ voltage-dependent channels, the landmarks of this group are the channels characterized by a delayed rectifier current (K_DR_). The “classical” K_DR_ shows fast-activating kinetics at a low-threshold level and is mediated by Kv1/KCNA family α subunits [42,65]. In particular, Kv1.1 activates rapidly upon small membrane depolarizations, while Kv1.2 requires stronger ones [66,67]. These latter are predominantly localized at the axon initial segment (AIS), the presynaptic terminal sites and juxtaparanodal regions of the nodes of Ranvier of medium and large myelinated axons [46,47,68,69]. Consistent with their neuronal distribution and kinetics, Kv1.1 and Kv1.2 have a pivotal role not only in limiting the action potential generation and propagation, but also in modulating the shape and the rate of action potentials and the neurotransmitter release [43,60,70]. Kv1.1 and Kv1.2 often combine with β subunits, such as Kvβ2, to form functional heteromeric K^+^ channels, whose trafficking and functions depend on the association with other accessory proteins [60,65,70]. Notably, some proteins with great relevance are the cell adhesion molecules Caspr2 and TAG-1, the cytoskeletal scaffold 4.1B and multiple members of the ADAM family that allow a different distribution along the axon areas [70,71].

In addition to the “classical” K_DR_, the M-type current (I_M_) has a certain relevance too. I_M_ shows slow-activating kinetics at a low-threshold level and is principally mediated by Kv7/KCNQ family α subunits [67,72,73]. Like the Kv1.1 subunit, K7 activation requires only small depolarizations, but it can even be activated at the resting potential. Moreover, Kv7 channel activity can be modulated by G-proteins associated with Muscarinic acetylcholine receptors, hence the origin of the I_M_ name [73,74]. Kv7 kinetics mirror its functions as it aims to maintain the resting potential and reduce neuronal excitability [43,52,72]. Consistent with their fundamental role, Kv7 subunits are selectively localized to the AIS and the nodes of Ranvier, where homomers of the Kv7.2 subunit or heteromers with Kv7.3 exclusively form functional channels [42,60,67]. As well as in the CNS, Kv7.2 and Kv7.3 are widely present in the PNS, where they play an important role in the nociceptive pathway. Indeed, an alteration of their currents may cause a strong neuronal hyperexcitability, leading to different physiopathologies such as epilepsy, peripheral sensitization and neuropathic pain [52,73,75,76].

### 2.3. Sodium–Calcium Exchanger (NCX) Family

The NCX exchangers (Ca^2+^/Na^+^) are members of a much larger family of transport proteins, the CaCA (Ca^2+^/cation antiporter) superfamily, which play a hallmark role in controlling the Ca^2+^ flux across the plasma membrane or between intracellular compartments [77,78]. The CaCA proteins possess a conserved sequence and share a similar topology that has been extensively studied in many organisms [79,80,81]. In particular, mammals express three different SLC8 (A1–A3) genes, which, respectively, encode three NCX (1–3) exchangers that share about 70% overall amino acid identity [77,81].

The NCX family has a general topology composed of 10 transmembrane domain segments (TMS) and a large intracellular regulatory f-loop between TMS5 and TMS6. The latter contains two calcium-binding domains (CBD1-2) and an XIP domain. CBD1-2 are regulatory domains required for intracellular ion sensing and binding; otherwise, the XIP domain consists of a small auto-inhibitory sequence that confers Na^+^-dependent inactivation. Moreover, there are two highly conserved repeats, α-1 and α-2, localized between TMS2-TMS3 and TMS7-TMS8, respectively, which form the ion transport regions [79,82,83,84]. At the post-transcriptional level, only NCX1 and NCX3 undergo alternative splicing of the primary nuclear SLC8A1 and SLC8A3 transcripts, and each variant exhibits distinct properties for Ca^2+^ sensing and fluxes [77,79].

NCX is a low-affinity high-capacity transporter that shows an electrogenic coupling ratio of 1:3 Ca^2+^/Na^+^ ions [85]. In particular, NCX mediates Ca^2+^ extrusion by combining the latter with the influx of Na^+^ ions, depending on the electrochemical gradient of each one under physiological conditions [85,86]. In addition to the forward mode just described, in some cases, NCX can contribute to Ca^2+^ influx into cells by operating in the reverse mode, coupling Ca^2+^ influx with Na^+^ efflux [86,87,88]. The latter mode is involved in the regulatory process of glutamatergic gliotransmission between astrocytes and neurons and in the NMDA/AMPA receptors’ activity, which produce Ca^2+^ entry [86,89]. Thus, NCX dysregulation plays a central role in subsequent Ca^2+^-related toxicity, which is strongly involved in the development of several diseases [86,87,90]. Furthermore, during both normal and pathophysiological conditions, NCX has emerged as a dominant mechanism for the Ca^2+^ efflux pathway and the protective regulation of cell homeostasis [77,91].

Consistent with its crucial role, NCX is present in both excitable and non-excitable tissues, such as brain, heart, kidney, pancreas and liver [79,92]. It is relevant that each splice variant is expressed in a tissue-specific manner, and only the brain shows significant expression of all three isoforms. Moreover, each variant has a unique cellular and subcellular distribution and probably a specialized functional role in Ca^2+^ homeostasis [83]. Although the NCX was discovered and extensively studied in cardiac myocytes, it has particularly important functions in both the CNS and the PNS, where excitable cells experience transient Ca^2+^ fluxes [85,93]. In particular, NCX1 is expressed ubiquitously, and it shows a great abundance in heart and brain, whereas NCX2 and NCX3 are more highly expressed in the PNS and skeletal muscle than NCX1 [92,94].

**NCX2**

In the CNS, NCX2 is involved in neurotransmitter release in both neuronal and glial cells with a relevant distribution in astrocytes where the NCX2 antibody strongly cross-reacted with a glial fibrillar protein [93,95]. Moreover, the overall high expression of NCX2 in brain could partly be the result of the ratio of astrocytes to neurons (4:1) [95]. Consistent with this distribution, it is broadly clear that NCX2 exerts its influence at the framework of a neuron–glia network [79]. Moreover, NCX2 is highly detected in the membranes of neuronal cell bodies and at the presynaptic level in a cerebral structure-dependent manner [93,94,96]. For example, NCX2 is the major isoform involved in the clearance of Ca^2+^ in presynaptic terminals of CA1 hippocampal pyramidal neurons, where it is essential for the control of synaptic plasticity, memory, learning and cognition [83,96]. In the PNS, NCX2 is localized within the cell bodies of small-diameter DRG neurons and throughout the entire length of neurites and neuritic tips [97]. More precisely, NCX2 is localized in epidermal free nerve endings and in mechanosensory nerve endings, which include nociceptors [97,98]. Here, NCX2 is co-expressed with Nav 1.6, Nav 1.7, Nav 1.8 and Nav 1.9, whose physiological activities influence each other in both normal and pathological conditions [86,91,98]. In this regard, Persson and colleagues demonstrated that the presence of NCX2 together with Na^+^ channels within epidermal nociceptive terminals may thus make these fibers especially sensitive to injury when energetically challenged [99]. Overall, NCX2 has an important role not only in regulating Ca^2+^ homeostasis but also in noxious stimulus transmission [86,91,97,99]. Thus, NCX2 exerts important antinociceptive effects, and its alteration is strongly related to peripheral sensitization and neuropathic pain [86].

**NCX3**

In the CNS, NCX3 is expressed at the lowest level of the three isoforms and may play a highly specialized role in Ca^2+^ homeostasis in certain cell types, such as small subfields of neurons involved in Long-Term Potentiation (LTP) [93,100]. Consistent with its distribution in hippocampal neuronal cell bodies as well as in the associated dendritic network, Molinaro and colleagues demonstrated that NCX3 impairment has important consequences for basal synaptic transmission, LTP regulation, spatial learning and memory performance [100]. Moreover, this isoform shows a particularly low expression level in astrocytes too [95]. Interestingly, NCX3 is the main isoform expressed in oligodendrocyte progenitor cells (OPC), where it has a critical role in oligodendrocytes’ maturation and in consequent neuronal myelination [90]. Thus, NCX3 dysfunction causes a relevant reduced size of hypo-myelinated spinal cord, where it is highly localized in both white and gray matter [83]. Unlike NCX1-2, NCX3 has the peculiar capability of maintaining Ca^2+^ homeostasis even when ATP levels are reduced significantly, highlighting its major role in neuronal preservation and protection during different pathophysiological conditions [90,100].

### 2.4. Voltage-Gated Ca^2+^ Channels (VGCCs or Cav)

Voltage-gated Ca^2+^ channels (VGCCs or Cav) are some of the most important regulators of Ca^2+^ concentration [101] that are under fine regulation in order to maintain it lower inside the cell (≈50–100 nM) than in the extracellular milieu (≈2 mM) [102]. Indeed, free Ca^2+^ is an important intracellular messenger in all cells, where it controls several cellular functions but can become toxic and cause cell death [103]. The homeostatic control of intracellular calcium concentration ([Ca^2+^]_i_) is maintained through the action of the plasma membrane Ca^2+^ transport ATPase (PMCA) and Na^+^/Ca^2+^ exchanger (NCX) in a resting cell. Upon elevated [Ca^2+^]_i_, Ca^2+^ is sequestered intracellularly by the sarcoendoplasmic reticulum Ca^2+^-ATPase (SERCA) and by the mitochondrial Ca^2+^ uniporter (mtCU), which are activated by Ca^2+^. Elevation of [Ca^2+^]_i_ can result from either the influx of extracellular Ca^2+^ or the release of Ca^2+^ from intracellular stores after various cell stimuli such as membrane depolarization, extracellular signaling molecules or intracellular messengers [104]. Since VGCCs are some of the main regulators of [Ca^2+^]_i_, they have an important role, especially in neurons, where, in addition to functions common to all cells, they also regulate cell excitability, and they have an important role in any neuronal function, including physiologic nociception and neuropathic pain [101].

#### 2.4.1. Topology and Subunits

VGCCs are heteromultimeric complexes consisting of a principal transmembrane pore-forming α_1_ subunit (190 kDa) that includes the structural and functional machinery required to conduct Ca^2+^ (Ca^2+^-selective pore, voltage sensor and gating mechanism) and a combination of three auxiliary subunits, a disulfide-linked α_2_δ dimer (170 kDa), an intracellular phosphorylated β subunit (55 kDa) and a transmembrane γ subunit (33 kDa), involved in the modulation of channel properties [105].

The α_1_ subunit is constituted by four homologous domains (D_1_–D_4_) connected to each other by three intracellular loops, respectively (L_1_–L_3_). Each domain consists of six transmembrane-spanning hydrophobic α-helical segments (S_1_–S_6_), and each segment is connected by two intracellular (between S_2_–S_3_ and S_4_–S_5_) and three extracellular linkers (between S_1_–S_2_, S_3_–S_4_ and S_5_–S_6_; the last ones are called P-loops). Both the N-terminus and C-terminus are intracellular [15].

The α_1_ subunit is the key molecule of the channel complex because it is capable of Ca^2+^ conduction, while the α_2_δ, β and γ subunits are auxiliary. It is known that some types of VGCCs are formed through the assembly of α_1_ subunits and ancillary ones (i.e., the high-voltage-activated channel; see below), while others appear to lack these ancillary subunits (i.e., the low-voltage-activated channel; see below) [9].

The α_2_δ subunit increases the maximum current density by increasing the α_1_ expression in plasmatic membrane together with reducing its turnover (exerts its effect on Ca^2+^ channel trafficking) and increasing also the inactivation rate [106]. It might have a role in modulating the excitability of DRG; indeed, the α_2_δ_1_ overexpression resulted in enhanced currents and altered biophysical properties in sensory neurons, supporting its contribution in neuropathic pain [107].

Similarly, the β subunit increases the maximum current density by causing a hyperpolarization shift in activation and by increasing the channel opening probability. It is not yet clear if its effect is also due to an enhancement of α_1_ trafficking [106].

Instead, the γ subunit, despite being known to bind the α_1_ D_4_ domain, has a controversial role as auxiliary subunit, and its functions are largely unknown [108].

VGCCs generally activate, inactivate and deactivate slower than VGSCs and can therefore be distinguished on a temporal basis [109].

#### 2.4.2. Classification, Physiological and Pharmacological Properties and Isoforms Involved in Neuropathic Pain

VGCCs are classified according to (i) their activation threshold or (ii) their amino acid sequence homology of the α_1_ subunit (Cav). In particular, VGCCs are classified in high-voltage-activated (HVA) and in low-voltage-activated (LVA) channels, and, at the molecular genetic level, are classified in three families (Cav1, Cav2 and Cav3) [109]. α_1_ subunits are currently classified into 10 subtypes (Cav1.1–Cav1.4, Cav2.1–Cav2.3 and Cav3.1–Cav3.3), of which 9 are expressed in the nervous system.

All 10 α_1_ subunits subtypes share a common transmembrane topology, but they have different biophysical properties (activation, inactivation, conductance and deactivation), expression patterns and pharmacology [102].

There is another classification according to Ca^2+^ currents that show distinct biophysical properties and that are differently modulated by pharmacological agents. For the different classes of Ca^2+^ currents, an alphabetical nomenclature has been adopted (L, N, P/Q, R and T type) [110].

HVA channels activate in response to strong depolarizations, and they generate long-lasting calcium influxes. They are further classified as L type (Cav1.1–Cav1.4; L for large and long-lasting), P/Q type (Cav2.1; P for Purkinje cells), N type (Cav2.2; N for neurons) and R type (Cav2.1; R for resistant) according to their different pharmacological sensitivity. L-type Ca^2+^ is blocked by dihydropyridines, phenylalkylamines and benzothiazepines (organic L-type Ca^2+^ channel antagonists). N-type, P/Q-type and R-type Ca^2+^ are blocked by specific polypeptide toxins from snail and spider venoms.

LVA channels, also classified as T type (Cav3.1–Cav3.3; T for tiny and transient), activate in response to weak depolarizations (between −65 mV and −50 mV), and they open transiently (under both brief and long depolarizations). They are resistant to the other VGCCs’ blockers. They are expressed in a wide variety of cell types, where they are involved in shaping the AP and controlling patterns of repetitive firing. T-type channels also generate the so-called window current, defined as small tonic inward current around resting membrane potentials, as a result of activation and inactivation curve overlap [109,110].

It has been shown that some VGCCs have greater involvement in pain pathways than others; among these, there are N-type and some T-type channels (Cav3.2) [111,112].

**Cav2.2 (N-Type Channel)**

Cav2.2 is exclusively expressed in the central and peripheral nervous systems including the brain, spinal cord and primary sensory neurons [102]. Its expression is particularly high in the superficial layer (laminae I and II) of the dorsal horn, which includes the major termination zone of nociceptive primary afferents [113]. Several studies demonstrated the implication of Cav2.2 channels in the transmission of pain signals at the spinal level. Indeed, it has been demonstrated that the pharmacological block of N-type channels stops the release of pro-nociceptive neurotransmitters such as glutamate and substance P [114]. Also, genetic models support its involvement, since Cav2.2^−/−^ mice showed markedly reduced neuropathic pain symptoms after spinal nerve ligation [115]. The N-type channel inhibitors have been suggested in the treatment of some forms of pain [116].

**Cav3.2 (T-Type Channel)**

Cav3.2 is expressed in all parts of the sensory neurons involved in the transmission of the nociceptive signal, including peripheral nerve endings, axons, soma and dorsal horn synapses [116]. It is known that T-type channels modulate cellular excitability and rhythmic activity, and that they are involved in pathophysiological conditions related to neuronal hyperexcitability [113]. Cav3.2 might have a role in the nociceptive pathway or in lowering the threshold for AP generation [117] or in enhancing Ca^2+^-dependent neurotransmitter release, which results in synaptic facilitation [118]. Its role in pain states is supported by findings that showed that antisense knockdown of Cav3.2, but not Cav3.1 and Cav3.3, channels in DRG neurons, resulted in marked antinociceptive, anti-hyperalgesia and anti-allodynia effects [119].

### 2.5. Transient Receptor Potential Family (TRPA1, TRPM8 and TRPV1)

Transient receptor potential (TRP) receptors are involved in the development of chemotherapy-induced peripheral neuropathic pain, which is a common side effect of selected chemotherapeutic agents such as oxaliplatin [120]. TRP channels have six transmembrane-spanning domains (S1–S6), with a pore-forming loop between S5 and S6, and both the C- and N-termini are located intracellularly [121]. TRPC channels are nonselective cation channels expressed in excitable and non-excitable cells [122]. TRPV channels are a part of the TRP channel superfamily and named for their sensitivity to vanilloid and capsaicin [123]. In most tissues, TRPV channels serve as sensors for different pain stimuli (heat, pressure and pH) and contribute to the homeostasis of electrolytes, the maintenance of barrier functions and the development of macrophages [124]. TRPA1 is the only TRPA protein present in humans. TRPA1 is a sensor for diverse noxious external stimuli such as intense cold, irritating compounds, mechanical stimuli, reactive chemicals and endogenous signals associated with cellular damage [125]. The TRPM subfamily consists of eight members. TRPM1–TRPM8.42 TRPMs are involved in several physiological and pathological processes. TRPM channels possess a large cytosolic domain, making them the largest members of the TRP superfamily [126]. The mucolipin family of the ion channel TRP superfamily (TRPML) includes three members: TRPML1, TRPML2 and TRPML3. Defects in TRPML function are predicted to have important effects on organelle acidification, vesicle fusion, endosome maturation and signaling, thus suggesting that this protein family plays a key role in normal and pathological conditions [127].

## 3. Ion Channels/Transporters in Chemotherapy-Induced Peripheral Neurotoxicity Models

First of all, studies are characterized by a vast heterogeneity in dosages/schedules of chemotherapy drugs as well as the outcome measures elected to detect/grade neurotoxicity, making comparing different studies complex. Moreover, a clear distinction should be made in particular for in vivo studies; not all studies actually demonstrated the onset of neuropathy but just a neuropathic pain/nocifensive behavior, making it arguable, in particular in the preventive setting, that an actual effect on neuropathy onset can be expected in a clinical setting. Keeping in mind these aspects, the following key information can be retained. Table 1 and Table 2 summarize in vitro and in vivo findings, respectively, to provide the reader with a broader overview of what is available in the literature; however, the majority of the data presented cannot be easily translated to the clinical setting since the dosage/schedule does not mirror the actual phenomenon observed in clinical practice. Therefore, while planning a study in this field, a careful evaluation of this aspect should be performed, as recently suggested by Cavaletti et al. in 2024 [128].

### 3.1. Voltage-Gated Sodium Channels

VGSCs were linked to peripheral neurotoxicity in in vitro studies after exposure to oxaliplatin (OHP). Adelsberger et al. [129] first observed that dorsal root ganglia (DRG) neurons exposed to OHP have an increase in Na^+^ currents by exploiting patch clamp recordings. Exploiting the same technique, alternations in this channel were demonstrated by multiple other authors, confirming the robustness of this observation [130,131,148] even though dosages/models vary among different studies. Verma et al. [132] tested VGSCs with micro/multielectrode array recordings, showing a possible hyperexcitability in neurons also after exposure to paclitaxel (PTX), and Nieto et al. [149] suggested that they might be involved in painful manifestations in PTX-treated animals.

In vivo studies confirmed involvement of VGSCs in OHP-related hyperexcitability [150], showing that, by decreasing this condition, neuroprotection can be established [151,152]. In particular, preliminary data suggest, in fact, that increased Na^+^ currents due to OHP exposure are able to activate the sodium–calcium exchanger (NCX) reverse mode, leading to Ca^2+^ neurotoxicity, as we will discuss in the subsequent sections [87].

### 3.2. Voltage-Gated Potassium Channels

A potential involvement of VGkCs was observed in the in vitro setting, in particular after exposure to cisplatin CDDP, leading to a reduction in K^+^ currents in DRG neurons, which were actually explored in a broader setting aiming to assess changes in Ca^2+^ homeostasis and related voltage-operated ion channels which were found to be altered [133].

In the in vivo setting, they were linked to painful manifestations due to PTX exposure [153,154]. Some authors also suggested that they might play a role in the neurotoxicity manifestations observed in rodent models exposed to OHP [155,156,157].

### 3.3. Sodium–Calcium Exchanger

As stated above, the NCX, and, specifically, the isoform NCX2, was suggested to be the actual link between acute OHP-related transient neurotoxicity (i.e., a state of axonal hyperexcitability that mainly lasts in patients 24–72 h after OHP exposure [173,174,175]) and the actual OHP-induced axonal damage. The hypothesis, corroborated by preliminary findings by Ballarini et al. [87], is that Na^+^ currents increased due to OHP exposure trigger NCX2 reverse mode, leading to Ca^2+^ toxicity. Both in vitro [134] and in vivo [158] studies suggested that this family could also play a role in PTX-related neurotoxicity.

### 3.4. Voltage-Gated Ca^2+^ Channels

Alterations in Ca^2+^-related currents were suggested in in vitro models after exposure to PTX [135], CDDP [136], OHP [137] and bortezomib (BTZ) [138]. In the in vivo setting, similar observations were made in relation to CDDP [136,159], PTX [135,160,161,162,163], BTZ [138,164] and vincristine (VCR) [165].

### 3.5. Transient Receptor Potential Family (TRPA1, TRPM8 and TRPV1)

In vitro findings suggest that this family could play a role in PTX-, OHP-, CDDP- and BTZ-related CIPN [139,140,141,142,143]. In vivo data support its role mostly in painful phenomena related to CIPN due to OHP, PTX, CDDP and BTZ [120,139,140,142,143,144,148,166,167,168].

## 4. Possible Clinical Translation

**Figure 2:**
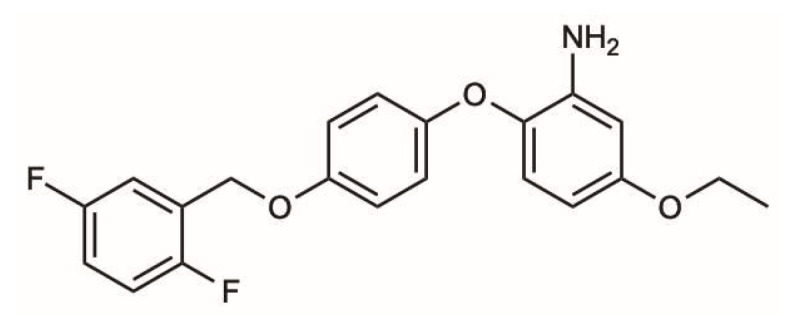
Molecular structure of SEA0400. SEA0400 is a novel and selective inhibitor of the Na^+^-Ca^2+^ exchanger (NCX), inhibiting Na^+^-dependent Ca^2+^ uptake.

The mechanism underlying CIPN remains unclear though mechanistic studies have reported that Na^+^, K^+^ and Ca^2+^channels and different types of transient receptor potential family are suggested to be involved [176]. As stated above, preclinical data should be carefully weighted to translate inferences from bench to bed side. As already stated, each study should be carefully evaluated considering schedule/dosages and outcome measures both in the in vitro and in vivo setting. For in vivo studies in particular, it should be pointed out that a robust CIPN animal model cannot rely just on behavioral tests; neuropathological and neurophysiological studies are warranted to test both large and small nerve fibers [177,178], which can be differently affected by different anticancer drugs [175]. Moreover, to translate data on voltage-operated ion channels from the in vitro to the in vivo setting, and then to the clinical setting, a specific method can be used: nerve excitability testing (NET) [179]. NET was first described in humans and then adapted to animal models [180,181]. A virtuous example of this is given by studies performed on OHP. Both in patients [182,183] and animal models [87,150,151], NET was able to demonstrate that OHP can induce a transient alteration that perfectly mirrors the expected pattern of a VGSC’s channelopathy. Starting from this observation, a pathogenetic hypothesis was then built, as already discussed, linking these phenomena (i.e., aberrant persistent Na^+^ currents) to NCX2 reverse mode activation. Rather promising neuroprotection data were obtained in animal models, targeting the modulation of this double Na^+^/Ca^2+^ axis, on the basis of strong outcome measures, as previously stated (i.e., neurophysiology and neuropathology to assess and grade axonal damage) [87], exploiting a selective modulator for the NCX family, such as SEA0400 (see Figure 2). The administration of sodium channel blockers was reported to be effective against oxaliplatin-induced neuropathic pain in humans [184] and animals [185], suggesting that concentrating on the voltage-gated sodium channel may be an effective treatment approach [151].

## 5. Concluding Remarks

**Figure 3:**
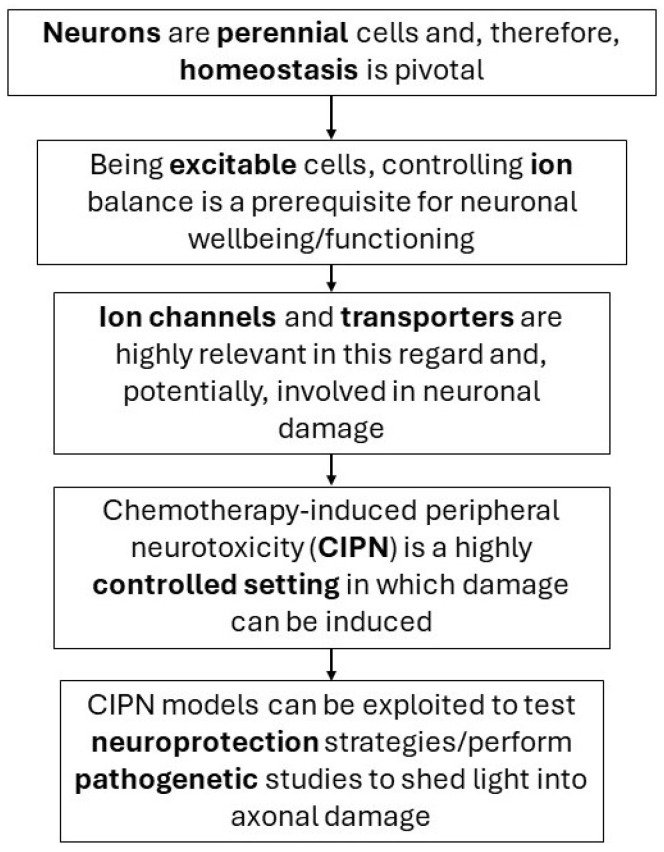
Graphical representation of the overarching hypothesis: targeting ion channels/transporters to prevent axonal damage.

Voltage-operated ion channels/transporters are clearly pivotal components of neurons, and it is not illogical to expect quite a vast involvement of these in CIPN axonal damage; therefore, our hypothesis is that, by targeting these specific elements, it is possible to potentially detect drugs/mechanisms to actually prevent axonal damage (see Figure 3). Already known or even known modulators can be tested, also exploiting the possibility of designing new drugs exploiting innovative approaches such as computational biology, as summarized in a clear-cut review by Azad et al. in 2023 [186].

Promising in vitro preclinical data should be carefully weighed based on the specific model used, as well as in vivo studies. Once robust data are available, they could be transferred to the bed side for neuroprotection trials, relying on highly translational outcome measures such as NET. Voltage-operated ion channels are an intriguing option in this setting since, when targeting them, the eventual neuroprotectant drug is unlikely to target the same mechanism that the anticancer drug exploits to obtain its oncological efficacy.

## Figures and Tables

**Table 1:** In vitro studies of Ion Channels/Transporters in Chemotherapy-Induced Peripheral Neurotoxicity Models.

Authors	Target	Cell Culture and Treatment	Neurotoxicity Assessment	Observations
Ballarini et al. [87]	NCX2	OHP 7.5 µM. Rat DRG neurons	Neurite elongation	Protection of neurite outgrowth with a selective NCX blocker
Adelsberger et al. [129]	VGSCs	OHP 250 µM. Rat DRG and hippocampal neurons	Patch clamp recordings	Increase in the Na^+^ current on DRG neurons but not on hippocampal neurons
Chang et al. [130]	VGSCs	PTX 0.1–1 µM. Human DRG neurons	Immunohistochemistry, qRT-PCR, transient Na^+^ currents and action potential frequency	Increase in Nav 1.7 mRNA expression but not Nav 1.8. Increased transient Na^+^ currents amplitude and action potential firing frequency
Lee et al. [131]	VGSCs	OHP 10 and 100 µM. Rat DRG neurons	Patch clamp recordings	Alteration of VGSC conductance towards negative membrane potentials in A-fibers of DRGs
Verma et al. [132]	VGSCs Nav 1.7 and Nav 1.8, KDR, KA, leak channel	PTX 250 nM. Rat DRG neurons	Micro/multielectrode array recordings	Decrease in PTX-induced hyperexcitability by a Nav 1.8 blocker and a KDR agonist treatments
Tomaszewski et al. [133]	VGCCs, VGKCs, VGSCs	CDDP 1, 5, 10, 50 and 100 µM. Rat DRG neurons	Patch clamp recordings	Decrease in Ca^2+^ and K^+^ currents in small DRG neurons but only a trend toward reduction in Na^+^ currents
Brenneman et al. [134]	mNCX-1	PTX 3 µM. Rat DRG neurons	Cell viability assays, IR cell bodies and neuritic areas	mNCX-1 siRNA decreases CBD protection from PTX toxicity, decrease in IR neuronal cell bodies and neuritic IR areas
Li et al. [135]	VGCC T-type	PTX 1 µM. Human DRG neurons	Patch clamp recordings, immunohistochemistry	Increase in Ca^2+^ current, increase in DRG excitability
Leo et al. [136]	VGCCs	CDDP 0.5 or 5 µM. Rat DRG neurons	Patch clamp recordings, immunostaining, calpain activity assay	Decrease in Ca^2+^ current in L-type, P-/Q-type and T-type channels but increase in N-type VGCC currents. Increased expression of N-type VGCC proteins. DRG neuroprotection by N-type VGCC blocker
Schmitt et al. [137]	VGCCs	OHP 1, 10, 100, 250 and 500 µM. Rat DRG neurons	Patch clamp recordings, immunocytochemistry, Western blot, calpain activity assay	Decrease in L-type, P/Q-type and T-type VGCCs currents. Prolonged treatment increased current density. Increase in L-type and T-type VGCCs protein expression. Increase in the action potential amplitude through modulation of T-type and L-type VGCCs
Tomita et al. [138]	VGCCs T type	BTZ 0.1 nM. Mouse neuroblastoma x rat DRG neuron hybrid cells	Western blot, qRT-PCR, patch clamp recordings	Increase in T-type VGCC protein expression. Increase in Ca^2+^ currents
Materazzi et al. [139]	TRPA1, TRPV4	PTX 10, 30 and 50 µM. Mice DRG neurons or esophagus slices	Ca^2+^ imaging, neuropeptides release assay	Modulation of TRPA1 and TRPV4 by Ca^2+^-dependent CGRP secretion
Nassini et al. [140]	TRPA1	OHP or CDDP 100 µM, guinea pig pulmonary artery. TRPA1+ CHO cells expressing mouse (10 to 300 µM OHP/CDDP)	Guinea pig pulmonary artery assay of neurogenic relaxation. DRG and CHO Ca^2+^ response to OHP or CDDP	OHP and CDDP activate TRPA1 channel on nociceptive nerve terminals. The activation of TRPA1 is mediated by oxidative stress
Sanchez et al. [141]	TRPV4	PTX 1 µM. Human SH-SY5Y cells	qRT-PCR, Western blot, patch clamp recordings, cytosolic Ca^2+^ measurement	Increase in TRPV4 protein and mRNA expression. Increase in outward and inward current density. Increase in cytosolic Ca^2+^ concentrations
Ta et al. [142]	TRPV1, TRPM8, TRPA1	CDDP or OHP 6.7 µM. Rat DRG neurons	qRT-PCR	TRPV1, TRPM8 and TRPA1 mRNA expressions are differently upregulated by CDDP and OHP
Trevisan et al. [143]	TRPA1	BTZ 10 or 100 µM. Mouse DRG neurons	Ca^2+^ imaging	BTZ did not evoke Ca^2+^ responses in TRPA1+ neurons
Ertilav et al. [144]	TRPV1	DT 10 nM. TRPV1 transfected SH-SY5Y cells	Ca^2+^ fluorescence, Western blot	Activation of TRPV1
Anand et al. [145]	TRPV1, TRPA1, TRPM8	OHP 12–120 µM. Rat DRG neurons	Neurite elongation and density, cell viability assay, cAMP assay, Ca^2+^ imaging	TRPV1 and TRPA1 sensitization but not for TRPM8
Leo et al. [146]	TRPA1, TRPV1	CDDP and OHP 10 µM. Rat DRG neurons	Cell viability assay, immunocytochemical staining, cytosolic and intramitochondrial Ca^2+^ measurement	Increase in cytosolic Ca^2+^ concentration and decrease in intramitochondrial Ca^2+^ concentration in TRPA1+ and TRPV1+ DRG neurons
Sanchez et al. [147]	TRPA1	PTX 1 µM. Human SH-SY5Y cells	qRT-PCR, Western blot, patch clamp recordings, cytosolic Ca^2+^ measurement	Increase in TRPA1 protein expression, TRPA1 current density and TRPA1-mediated Ca^2+^ concentrations

AMP: cyclic adenosine monophosphate; ASIC: acid-sensing ion channel; BTZ: bortezomib; CAP: compound action potential. CBD: cannabidiol; CDDP: cisplatin; CGRP: calcitonin gene-related peptide; CHO: Chinese hamster ovary; CMAP: compound muscle action potential; DT: docetaxel; HCN: hyperpolarization-activated cyclic nucleotide gated; IENFD: intraepidermal nerve fiber density; GBP: gabapentin; GIRK: G-protein-gated inward rectifier K+ channel; IR: immunoreactive; K2p1.1: potassium channel subfamily K member 1; KDR: delayed rectifier potassium channel; KA: A-type transient potassium channel; MOR: μ-opioid receptor; NCS: nerve conduction studies; NCV: nerve conduction velocity; NCX: sodium–calcium exchanger; NET: neuronal excitability testing; OHP: oxaliplatin; P2 × 3: purinergic receptor; PTX: paclitaxel; SNAP: sensory nerve action potential. TG: trigeminal ganglia; TREK: TWIK-related K+ channel; TRP: transient receptor potential channels, vanilloid subtype; TRPM: transient receptor potential melastatin; TRPA: ankyrin-type transient receptor potential; TTX: tetrodotoxin; VCR: vincristine; VGCCs: voltage-gated calcium channels; VGSCs: voltage-gated sodium channels; VGKC: voltage-gated potassium channel.

**Table 2:** In vivo studies of Ion Channels/Transporters in Chemotherapy-Induced Peripheral Neurotoxicity Models.

Authors	Target	Animal Model	Neurotoxicity Assessment	Observations
Ballarini et al. [87]	NCX2	OHP 7 mg/kg in mice, i.v., once a week for 8 weeks	NCS and NET recordings, mechanical allodynia test, immunohistochemistry, Western blot, caudal nerve morphology and morphometry, IENFD	Decrease in NCX2 protein expression in DRGs
Chukyo et al. [120]	TRPV1, TRPA1, TRPM8	OHP 6 mg/kg in rats, single i.p.	Acetone spray test, immunohistochemistry, in situ hybridization	Increase in TRPA1, TRPV1 and TRPM8 protein expression in DRGs. Increase in TRPA1 and TRPV1 mRNA coexpression in DRGs
Caudle and Neubert [148]	HCN, VGSCs, menthol, TRPM8	OHP 10 mg/kg in mice, i.p., two administrations; PTX 26 mg/kg in mice, i.p., four administrations. Dissociated TRG neurons *	Orofacial Pain Assessment Devices, patch clamp recordings	Increase in HCN, VGSCs and menthol evoked TRPM8 currents but not of VGKCs
Nieto et al. [149]	VGSCs TTX sensitive	PTX 2 mg/kg in mice, i.p., 5 days	Heat hyperalgesia test, acetone cold allodynia test, mechanical allodynia test, rotarod test	Decrease in heat hyperalgesia, mechanical and cold allodynia by TTX administration
Makker et al. [150]	VGSCs and VGKCs	OHP 10 mg/kg i.p. or 7.5 and 15 mg/kg i.m. in mice, single dose; 5 mg/kg i.p. on days 0, 2, 4, 6	CMAP and SNAP recording, mathematical modeling of axonal excitability	Change of the depolarization phase and creation of afterdischarges, inactivation of VGCCs, reduction in fast K^+^ conductance in motor axons. Increase in hyperpolarization and decrease in peak amplitude in sensory axons
Alberti et al. [151]	VGSC	OHP 5 mg/kg in rats, twice a week for 4 weeks	NCS and NET recordings, mechanical allodynia test, caudal nerve morphology and morphometry, IENFD	Modulating VGSC with topiramate (100 mg/kg per os, daily, starting 5 days before first OHP administration and continuing up to chemotherapy completion) complete neurotoxicity prevention was observed via neurophysiology, neuropathology and behavioral tests
Braden et al. [152]	VGSC Nav 1.7	OHP 3 mg/kg in mice, i.p., on days 0–4 and 10–14	Von Frey test	Decrease in mechanical allodynia through indirect inhibition of Nav 1.7
Di Cesare Mannelli et al. [153]	VGKCs Kv7	PTX 2 mg/kg in mice, i.p., on days 1, 3, 5 and 7; OHP 2.4 mg/kg in mice, i.p., on days 1–2, 5–9, 12–14	Cold plate test, Von Frey test, hot plate test	Kv7 channel blocker XE991 antagonized the pain-relieving activity of H2S donors, demonstrating the role of Kv7 in neuropathic pain
Jia et al. [154]	K^+^ channel 1.1 (K2p 1.1)	PTX 4 mg/kg in rats, i.p., every other day for a total of four injections, on days 0, 2, 4, and 6	Mechanical allodynia heat, heat hyperalgesia test and cold hyperalgesia test	Reduction in K^+^ channel 1.1
Kagiava et al. [155]	VGKCs	OHP 25, 100 and 500 µM. Rat isolated sciatic nerve *	Evoked CAP recordings	Induce alterations in CAP waveform, firing frequency and repolarization phase through VGKCs but not VGSCs
Kanbara et al. [156]	GIRK1	OHP 2 mg/kg in rats, i.p., twice a week for 4 weeks	Von Frey test	GIRK1 activation contributes to MOR antinociception
Lucarini et al. [157]	VGKC Kv7	OHP 2.4 mg/kg in mice, i.p., on days 1–2, 5–9 and 12–14	Cold plate test	Modulating Kv7 channels, a reduction in painful features is observed
Yilmaz et al. [158]	NCX	PTX 2 mg/kg in rats, on days 0, 2, 4 and 6. Dissociated DRG neurons *	Ca^2+^ imaging	PTX-induced inhibition of Ca^2+^ transients is not modulated by NCX activity
Li et al. [135]	VGCC T type	PTX 2 mg/kg in rats, i.p., on days 0, 2, 4 and 6	Von Frey test, patch clamp recordings, Ca^2+^ imaging, immunohistochemistry, Western blot	Increase in Ca^2+^ current, increase of DRG excitability, increase in T-type VGCC expression in DRGs and spinal cord. Decrease in mechanical allodynia by T-type VGCC blocker
Leo et al. [136]	VGCCs	CDDP 1.5 mg/kg in rats, i.p., two cycles of four daily administrations with four days rest	Von Frey test, hot plate test, rotarod test, Western blot, qRT-PCR	Increased expression of N-type VGCC proteins, but not mRNA in DRGs. Decrease in thermal hyperalgesia and mechanical allodynia by N-type VGCC blocker
Tomita et al. [138]	VGCCs T type	BTZ 0.4 mg/kg in mice, i.p., six administrations in 2 weeks	Western blot, Von Frey test	Increase in T-type VGCCs’ protein expression in DRGs. Decrease in mechanical hyperalgesia through T-type VGGC blockers and gene silencing
Nodera et al. [159]	Kv7 VDKCs	CDDP 2.3 mg/kg in mice, i.p., on days 1–5 and 13–17	SNAP recording, NET recording, NCS recording	Axonal protection, preserved membrane potential through increase in K^+^ currents with treatment Kv7 agonist retigabine
Kawakami et al. [160]	VGCCs	PTX 2 and 4 mg/kg in rats, i.p., on days 0, 2, 4 and 6. Dissociated DRG neurons	Von Frey test, patch clamp recordings	Increase in Ca^2+^ currents. GBP, a Ca^2+^ channel blocker, reverses mechanical hyperalgesia
Matsumoto et al. [161]	VGCCs α2δ-1 subunit	PTX 4 mg/kg in mice, single i.p. or i.v., or i.p. on days 0, 2, 4 and 6	Heat hyperalgesia test, electrical hyperalgesia test, qRT-PCR, Western blot, immunohistochemistry	Increase in DRGs’ expression of α2δ-1 subunit. GBP blockade of VGCCs decreases mechanical allodynia and sensitization of myelinated A-fibers
Okubo et al. [162]	VGCCs T type	PTX 2 mg/kg in rats, i.p., on days 0, 2, 4 and 6	Paw pressure test	Decrease in hyperalgesia through administration of T-type VGCCs selective blockers
Sekiguchi et al. [163]	T-type VGCCs	PTX 4 mg/kg in mice or 2 mg/kg in rats, i.p., on days 0, 2, 4 and 6	Von Frey test, paw pressure test, open field test, rotarod test	T-type VGCC blockers reduce neuropathic mechanical allodynia
Meregalli et al. [164]	VGCCs T type	BTZ 0.2 mg/kg in rats, i.v., three times a week for 4 weeks	NCV measurement, mechanical allodynia test, β-tubulin polymerization assay, IENFD, proteasome inhibition assay	Suvecaltamide modulation of T-type VGCCs reverses NCV and IENFD neuropathy, reverses β-tubulin polymerization increase but does not affect proteasome inhibition by BTZ
Sharma et al. [165]	VGCCs L type	VCR 50 μg/kg in rats, i.p., 10 days administration	acetone drop test, pin-prick test, hot plate test	Decrease in the protective effect of VCR pretreatment on allodynia and hyperalgesia following treatment with T-type VGCC blocker
Materazzi et al. [139]	TRPA1, TRPV4	PTX 6 mg/kg in WT and TRPA1 KO mice, single i.p.	Von Frey test, acetone cold stimulation test	Decrease in mechanical allodynia by TRPA1 and TRPV4 blockers. Decrease in cold hypersensitivity by TRPA1 but not TRPV4 blocker
Nassini et al. [140]	TRPA1	OHP 2 mg/kg i.v., CDDP 2 mg/kg i.p. in C57/BL6, Trpa1^+/+^ or Trpa1^−/−^ mice	Von Frey test, cold plate test, qRT-PCR	TRPA1 modulation decreases painful features related to OHP and CDDP single administration
Ta et al. [142]	TRPV1, TRPM8, TRPA1	CDDP 2.3 mg/kg or OHP 3 mg/kg in WT or TRPV1 KO mice, i.p., 5 days, 5 days rest and 5 days treatment	Von Frey test, radiant heat test, cold plate test, tail immersion test, qRT-PCR, immunohistochemistry	Upregulation of TRPV1 and TRPA1 mRNA following CDDP treatment, but only TRPA1 upregulation following OHP treatment in TGs. Decrease in mechanical allodynia following CDDP and OHP treatment in TRPV1 KO mice. Decrease in CDDP-induced thermal hypersensitivity in TRPV1 KO mice
Trevisan et al. [143]	TRPA1	BTZ 0.2, 0.5 or 1 mg/kg in WT or TRPA1 KO mice, single i.p.	Von Frey test, hot plate test, cold stimulation, chemical hyperalgesia test, rotarod test, Western blot	BTZ did not modify TRPA1 expression level in DRGs. Decrease in mechanical and cold hyperalgesia through TRPA1 agonist treatment and in TRPA1 KO mice
Chen et al. [166]	TRPV1, TRPV4, TRPA1	PTX 1 mg/kg in mice, i.p., on days 0, 2, 4 and 6	Von Frey test, hot plate test, cold hyperalgesia test	Reduction in heat hyperalgesia, but not mechanical allodynia and cold hyperalgesia, through TRPV1 blocking. Reduction in mechanical and heat, but not cold, hyperalgesia through TRPV4 blocking. Reduction in mechanical allodynia, heat and cold hyperalgesia through TRPA1 blocking
Ertilav et al. [144]	TRPV1	DT 30 mg/kg in mice, single i.p. Dissociated DRG neurons *	Von Frey test, hot plate test, Western blot, patch clamp recordings, Ca^2+^ fluorescence, cell viability assay	Increase in cytosolic Ca^2+^ concentration through TRPV1 channel agonist stimulation. Increase in TRPV1 expression level
Hori et al. [167]	TRPV1, TRPV2, P2 × 3 and ASIC3	CDDP 3 mg/kg in rats, i.p., once per week for five weeks	Von Frey test, pin-prick test, mechanical allodynia test, grid force test, rotarod test and immunohistochemistry	Increase in TRPV2, P2 × 3 and ASIC3 expression, but not in TRPV1 in DRGs
Quartu et al. [168]	TRPV1	BTZ 0.20 mg/kg in rats, single i.v., or three administrations for 8 weeks	Caudal NCV recordings, mechanical allodynia test, thermal hyperalgesia test, immunohistochemistry, morphometry, qRT-PCR and Western blot	Reduction in caudal NCV, increase in mechanical allodynia but not of thermal hyperalgesia. Increase in TRPV1 protein expression, but decrease in TRPV1 and CGRP mRNA level, in DRGs and spinal cord
Mao et al. [169]	K2p1.1 channel	PTX 4 mg/kg in mice, i.p., on days 0, 2, 4 and 6	Von Frey test, heat hyperalgesia, conditioned place preference, patch clamp recordings, qRT-PCR, Western blot, immunohistochemistry	PTX induces a decrease of K2P1.1 expression, contributing to chemotherapy-induced neuropathic pain
Pereira et al. [170]	TREK2	OHP 6 mg/kg in WT and TREK2 KO mice, single i.p.	Von Frey test, flinch test, immersion tests, hot plate test, thermal preference test, dynamic cold plate test; qRT-PCR; single C-fibers recordings	Decrease in TREK2 expression in DRGs. TREK2 mediates neuropathic hyperalgesia, regulates heat sensitivity of C-fibers, but does not play a role in noxious thermal hypersensitivity
Rapacz et al. [171]	VGSCs and L-type VGCCs	OHP 10 mg/kg in mice, i.p.	Von Frey test, hot plate test, formalin test	Decrease in mechanical allodynia by VGSCs and VGCCs blocking
Salat et al. [172]	VGSCs	OHP 10 mg/kg in mice, single i.p.	Von Frey test, cold plate tests, rotarod test	Reduced mechanical allodynia following a VGSC blocker

AMP: cyclic adenosine monophosphate; ASIC: acid-sensing ion channel; BTZ: bortezomib; CAP: compound action potential. CBD: cannabidiol; CDDP: cisplatin; CGRP: calcitonin gene-related peptide; CHO: Chinese hamster ovary; CMAP: compound muscle action potential; DT: docetaxel; HCN: hyperpolarization-activated cyclic nucleotide gated; IENFD: intraepidermal nerve fiber density; GBP: gabapentin; GIRK: G-protein-gated inward rectifier K^+^ channel; IR: immunoreactive; K2p1.1: potassium channel subfamily K member 1; KDR: delayed rectifier potassium channel; KA: A-type transient potassium channel; MOR: μ-opioid receptor; NCS: nerve conduction studies; NCV: nerve conduction velocity; NCX: sodium–calcium exchanger; NET: neuronal excitability testing; OHP: oxaliplatin; P2 × 3: purinergic receptor; PTX: paclitaxel; SNAP: sensory nerve action potential. TG: trigeminal ganglia; TREK: TWIK-related K^+^ channel; TRP: transient receptor potential channels, vanilloid subtype; TRPM: transient receptor potential melastatin; TRPA: ankyrin-type transient receptor potential; TTX: tetrodotoxin; VCR: vincristine; VGCC: voltage-gated calcium channels; VGSC: voltage-gated sodium channels; VGKC: voltage-gated potassium channel. * ex vivo studies.
